# Fibroadenoma in axillary accessory breast tissue: a case report

**DOI:** 10.1186/s13256-022-03540-2

**Published:** 2022-09-08

**Authors:** Ermias Teklehaimanot Yefter, Yitagesu Aberra Shibiru

**Affiliations:** 1 Department of Pathology, University of Gondar, Gondar, Ethiopia; 2 Department of Surgery, University of Gondar, Gondar, Ethiopia

**Keywords:** Axilla, Accessory breast tissue, Axillary accessory breast tissue, Fibroadenoma, Case report

## Abstract

**Background:**

Accessory breast(s) is defined as the presence of more than two breasts with or without a nipple and areola in human beings. It may occur anywhere along the primitive embryonic milk lines, which extend from the axilla to the groin. Accessory breast tissue can potentially undergo the same physiological and pathological processes as the normally located breast, including lactational change, fibroadenoma, and carcinoma. Although common in the normally located breast tissue, the incidence of fibroadenoma in accessory breast tissue is rare. Furthermore, if the swelling occurs in the axilla or groin, it may present a diagnostic challenge by clinically mimicking a lymphoma or other causes of lymphadenopathy. Owing to its rarity and its tendency to pose a clinical diagnostic challenge, we decided to report a case of fibroadenoma in axillary accessory breast.

**Case presentation:**

A 28-year-old Ethiopian female patient came to University of Gondar comprehensive specialized hospital with a complaint of left axillary swelling of 3 years duration. There was no history of cough, fever, weight loss, or night sweating. On physical examination, there was an approximately 5 × 4 cm, firm, well-defined, mobile, nontender solitary mass in the left axilla that was completely separated from the left breast. Fine-needle aspiration cytology suggested a diagnosis of fibroadenoma in axillary accessory breast tissue. The mass was completely excised, and histopathologic examination confirmed the diagnosis. Her recovery was uneventful. She was informed about the diagnosis, reassured, and discharged from care.

**Conclusion:**

In the clinical evaluation of a patient with axillary swelling, accessory breast tissue disorders such as fibroadenoma must be considered as a differential diagnosis for early diagnostic workup and management. Moreover, this case underscores the fact that, similar to normal breast tissue, accessory breast tissue is also susceptible to the same pathologic disease processes including neoplasms such as fibroadenoma.

## Background

Mammary gland (breast) development begins in the early embryonic period as ectodermal thickening. During the 6th week of embryonic development, the mammary milk lines, which represent two ectodermal thickenings, develop along the sides of the embryo, extending from the axillary region to the groin [1]. This line represents the location of the embryonic mammary ridges. In normal development, most of the embryologic mammary ridges along the milk line involute, except for two segments in the pectoral region, which later become breasts. When any of these ridges fail to involute, accessory breast develop. This is synonymous to supernumerary breast, ectopic breast, and polymastia, and usually occurs along the “milk line” or mammary line [2–4]. The prevalence of accessory breast tissue has been shown to be dependent on various factors, including gender, geographical area, race, and inheritance. On average, occurrence ranges from 0.22% to 6% of the general population [5].

Although fibroadenoma is a common benign lesion of normal breast tissue, its occurrence in accessory breast tissue is very rare and only few cases have been reported in the literature. They are clinically significant as they are associated with other congenital anomalies of the urinary and cardiovascular systems [6, 7]. Furthermore, if the swelling occurs in the axilla or groin, it may clinically mimic other tumorous lesions such as lymphoma or other causes of lymphadenopathy. Therefore, depending on their anatomic location, they can pose a diagnostic challenge to clinicians [8].

Herein, we report a case of fibroadenoma in the axillary accessory breast that was clinically considered as a lymphoma. We are reporting this case of uncommon occurrence owing to its rarity as a seat of origin in this anatomic location. Additionally, as is evident in our case presentation, the condition can pose a diagnostic challenge to clinicians by resembling lymphoma or other causes of lymphadenopathy. As a result, we report the case to emphasize the importance of considering accessory breast as well as its associated pathology in the differential diagnosis of axillary mass. Moreover, we also need to recognize the importance of evaluating the patients to rule out renal or cardiovascular anomalies, as these have an important association.

## Case presentation

A 28-year-old Ethiopian female came to University of Gondar comprehensive specialized hospital, northwestern Ethiopia, with a complaint of left axillary swelling of 3 years duration. The swelling was small initially and progressively increased in size to attain the current size. She had visited a nearby health center on multiple occasions for this complaint and took unspecified antibiotics, but experienced no improvement. Apart from the local mild pain and discomfort, there was no history of cough, fever, weight loss, night sweating, or other constitutional symptoms. She has no family history (first-degree relatives) of diabetes, hypertension, or any other remarkable noncommunicable disease including cancer. Her past medical history is not significant, except for occasional mild dyspeptic symptoms; she took unspecified over-the-counter anti-acids a couple of times for her dyspepsia as the symptoms arose. She has no history of admission to hospital. She has no history of any form of surgical procedure. She has no history of smoking. Except for the mild-to-moderate amount of local alcohol beverages she consumed on holidays, she has no history of excess alcohol consumption. She is a janitor in a public school. She is unmarried and lives with her parents. She has a fiancé and uses a combination of natural calendar method and male condom for contraception. She has no history of oral contraceptive pill (OCP) or injectable or intrauterine contraceptive device (IUCD) use. She has no history of pregnancy. She reported that she has regular menstrual cycles and no significant past obstetric or gynecologic history.

**Fig. 1:**
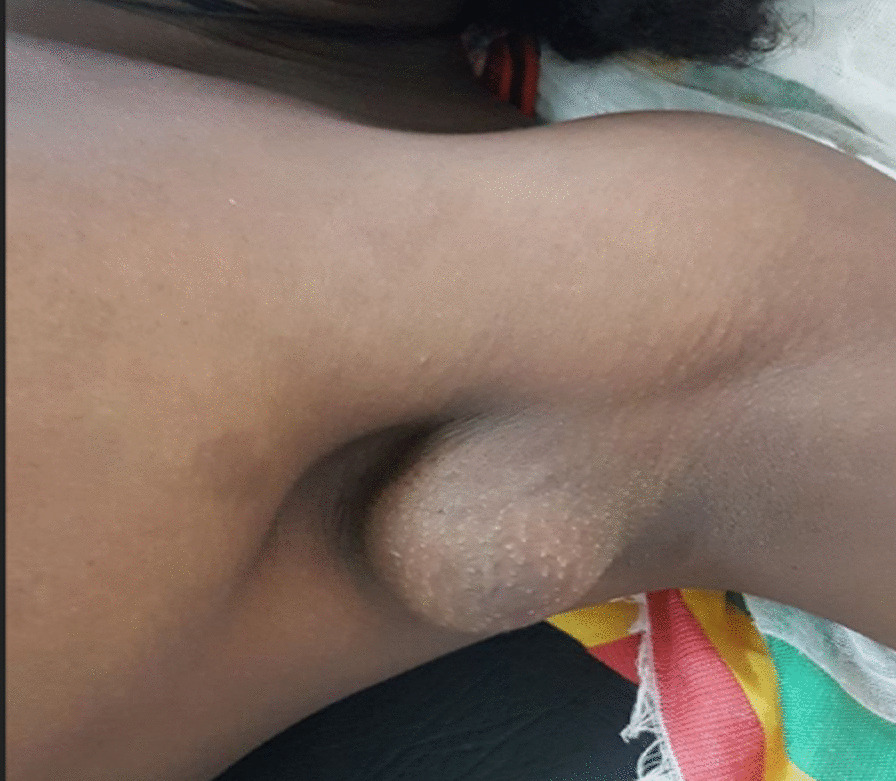
Firm nodular mass in the left axilla

On physical examination, her general appearance was stable, healthy looking, and ambulating. Vital signs were as follows: pulse rate, 82 beats per minute; blood pressure, 110/80 mmHg; respiratory rate, 18 breaths per minute; temperature, 36.2 °C. She has pink conjunctiva, non-icteric sclera, and no abnormal finding on head and neck examination. On lymphoglandular system (LGS) examination, there was an approximately 5 × 4 cm solitary mass in the left axilla (Fig. 1). It was firm in consistency, nontender, well defined, mobile, and completely separated from the left breast. Skin over the mass was normal with no nipple, areola, or ulceration. There were no clinically significant findings detected on examination of breasts, right axilla, or the other externally accessible lymph node regions. On chest examination, it was symmetrical and moved with respirations, and clear breath sounds were heard on auscultation. On precordial and cardiovascular examinations, the normal *S*_1_ and *S*_2_ heart sounds were heard, there was no murmur or gallop, and all the peripheral arterial pulses were palpable with strong and regular pulse. Abdomen was soft to palpation. No hepatomegaly, splenomegaly, or mass was detected, and there were no signs of fluid collection. No costovertebral angle tenderness or abnormal finding was detected on genitourinary system (GUS) examination. No pedal or other subcutaneous edema was identified on musculoskeletal examination. No palmar pallor or skin lesions were seen on integumentary system examination. On neurologic examination, she was conscious, alert, and fully oriented to time, place, and person. Cranial nerve examination revealed normal findings. No motor or sensory deficit was detected, and deep tendon reflexes were normal. On the basis of the above findings, a provisional clinical impression of axillary lymphadenopathy due to either lymphoma or tuberculosis was considered.

Laboratory investigations done on the same day of her presentation including complete blood count (CBC), erythrocyte sedimentation rate (ESR), and chest X-ray were noncontributory. On CBC, white blood cell (WBC) count was 5900/mL with 53% granulocytes, 42% lymphocytes, 1% eosinophils, and 4% monocytes. Platelet count was 247,000/mL. Hemoglobin was 13.4 g/dL with mean corpuscular volume (MCV) of 89 fL. ESR was 13 mm/hour. Renal function test revealed blood urea nitrogen (BUN) of 13 mg/dL, and serum creatinine level was 0.78 mg/dL. On liver function test, total bilirubin was 0.7 mg/dL, serum albumin was 4.4 g/dL, and serum aspartate transaminase (AST/SGOT) and serum alanine transaminase (ALT/SGPT) were 29 and 33 IU/L respectively. All of these laboratory test results were within the normal reference range of our laboratory. Urinalysis was also done, and it was normal.

**Fig. 2:**
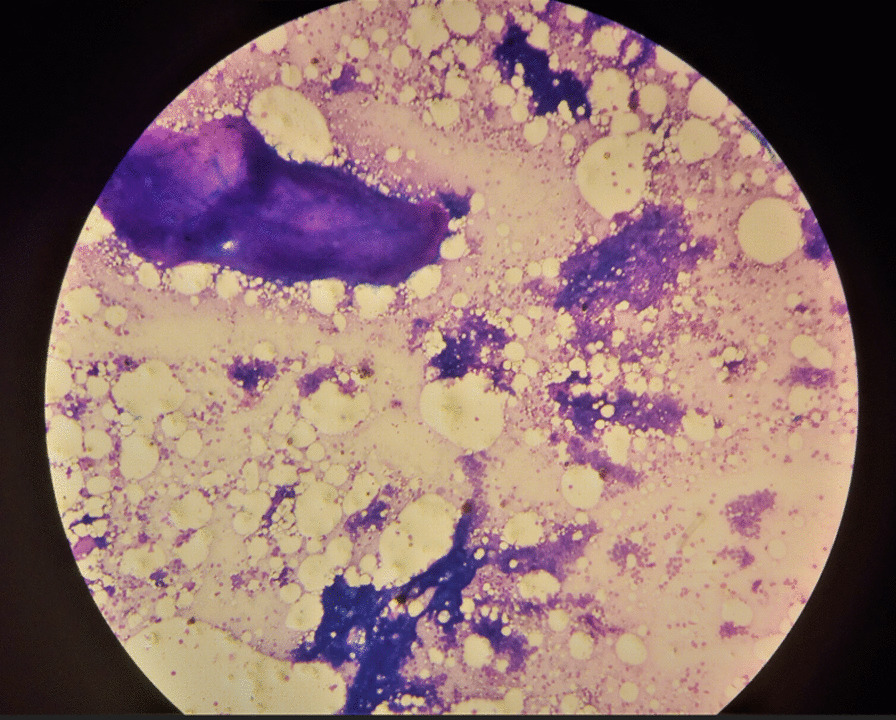
Low-power microscopy of fine-needle aspiration cytology from the mass showing tight cohesive clusters of bland ductal cells in branching pattern, fibromyxoid stromal fragments, and numerous scattered bare nuclei on the background (Wright’s stain)

Owing to the limited number of pathologists and long waiting list of patients, she was scheduled for fine-needle aspiration cytology (FNAC). Two weeks after her initial presentation, FNAC from the mass was done and smears were stained with Wright’s stain following the standard procedures. Microscopic examination revealed cellular aspirates of tight cohesive clusters of bland ductal cells in branching stag-horn-like pattern along with numerous scattered bare nuclei on the background and fibromyxoid stromal fragments (Fig. 2). Lymphoid cells were not seen. Fibroadenoma (in axillary accessory breast tissue) was suggested on the basis of these FNAC features. Abdominopelvic ultrasound and echocardiography were normal.

After discussion with the patient, we agreed to undergo surgical intervention for multiple indications, including discomfort, difficulty reaching full range of shoulder movement, cosmetic and hygienic reasons, and patient request. We opted to perform lumpectomy of the left axillary mass under general anesthesia. After securing an intravenous line and putting her on maintenance fluid, ketamine 30 mg plus diazepam 10 mg was prepared and administered intravenously. After light sedation, we infiltrated the skin incision area with 4 mg/kg lidocaine, and then underwent the lumpectomy under all aseptic precautions. The intraoperative finding was a firm, mobile, well-capsulated solitary gray-whitish 5 × 4 × 4 cm mass in the left axillary fossa. The mass was located superficially in the subcutaneous tissue separated from the left breast parenchyma. Postoperatively, we put her on intravenous diclofenac 50 mg, which was later switched to 100 mg is taken orally when needed (PO PRN) discharge. As she had a smooth postoperative course, she was discharged on the same day with appointment scheduled for follow-up and wound care. The wound healing process was uneventful, and she only took the antipain medication for only a couple of days.

**Fig. 3:**
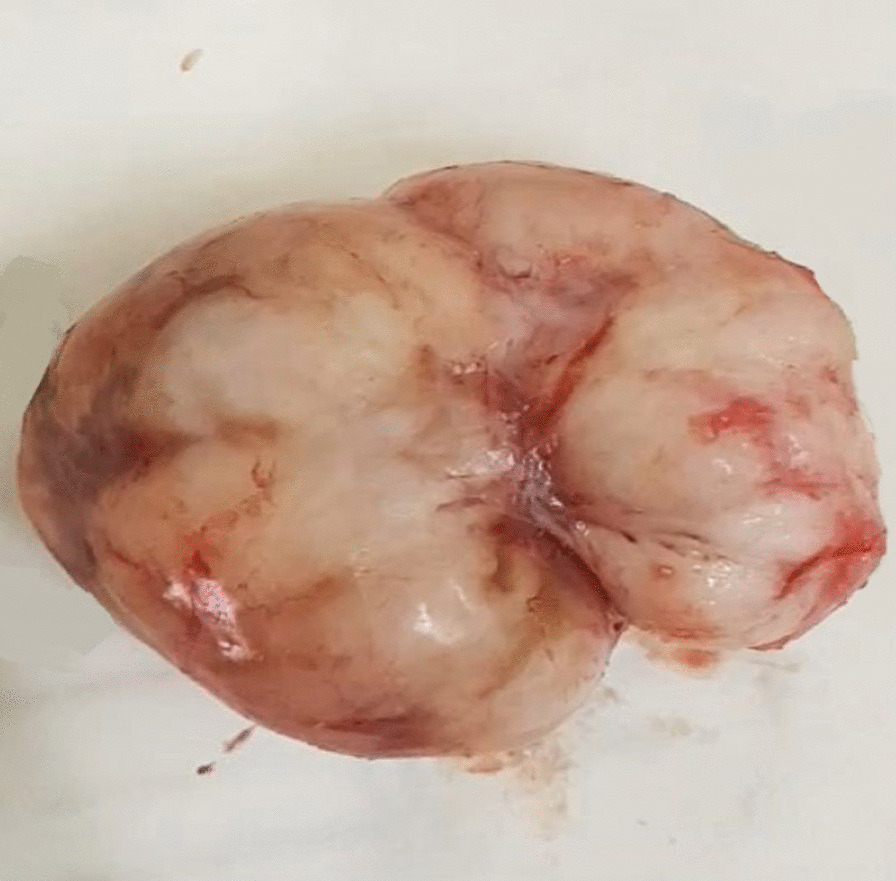
Excised tissue that is nodular and well circumscribed

**Fig. 4:**
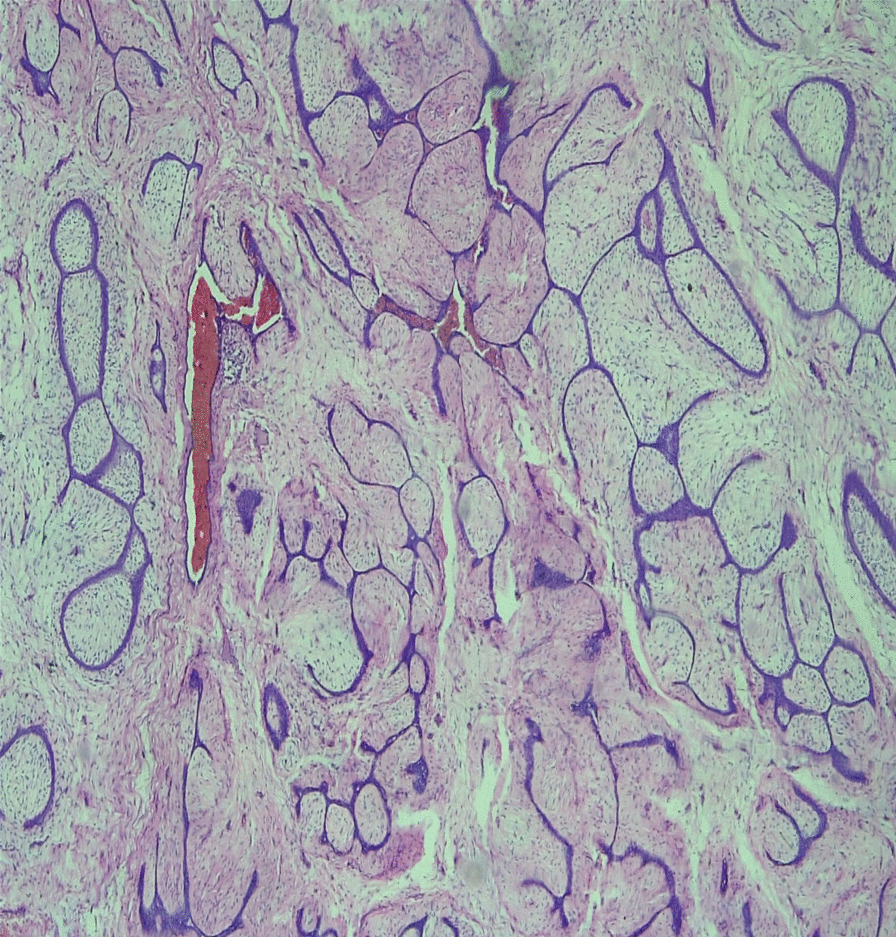
Low-power microscopy showing histopathologic features of a fibroadenoma

The excised mass was subjected to histopathologic evaluation following the standard tissue processing and staining procedure. Gross and microscopic histopathologic examination confirmed the diagnosis of fibroadenoma in axillary accessory breast (Figs. 3, 4). The histopathology report was issued 3 weeks after the day of her operation as there was a long biopsy waiting list and few pathologists were available in the hospital. She was informed about the pathology result, reassured, and discharged from care after 3 months of follow-up. The difficulty of shoulder movement and discomfort associated with the mass as well as other complaints were relieved. She was very much satisfied with the intervention and care given.

## Discussion

In this report, we presented a case of fibroadenoma in the axillary accessory breast in a 28-year-old female after she presented with a chief complaint of left axillary swelling of 3 years duration. The initial clinical impression was an axillary lymphadenopathy secondary to lymphoma to rule out tuberculosis. The diagnosis of fibroadenoma in the axillary accessory breast tissue was suggested by FNAC and later confirmed by histopathology after excisional biopsy. Although fibroadenoma is a common benign lesion of normal breast tissue, its occurrence in accessory breast tissue is very rare and only few cases have been reported in the literature. Furthermore, if the swelling occurs in the axilla or groin as seen in the current case report, it may clinically mimic other tumorous lesions such as lymphoma or other causes of lymphadenopathy. Therefore, as evident in our case presentation, depending on their anatomic location in the milk line, they can pose a diagnostic challenge to clinicians. In addition to its rarity and its potential to pose a diagnostic challenge to physician, we present this case report along with brief literature review to underscore the importance of evaluating such patients to rule out renal or cardiovascular anomalies as it has an important association.

Two hypotheses have been proposed on the development of accessory breast. One attributes the condition to the failure of involution of the milk line, while the other believes it develops from the modified apocrine sweat glands [9, 10]. The first one is the most widely known hypothesis and is linked to the embryogenesis of normal breast. Breast development begins in the early embryonic period as ectodermal thickening. During the 6th week of embryonic development, the mammary milk lines, which represent two ectodermal thickenings, develop along the sides of the embryo, extending from the axillary region to the groin [1]. This line represents the location of the embryonic mammary ridges. In normal development, most of the embryologic mammary ridges along the milk line involute, except for two segments in the pectoral region, which later become breasts. Accessory breast (ectopic breast/ supernumerary breast) develops when any of these extrapectoral mammary ridges fails to involute. Therefore, on the basis of this hypothesis, accessory breast may occur anywhere along the primitive embryonic milk lines. In support of this hypothesis, epidemiologically, the majority of accessory breast occurs in the milk line. However, atypical localizations are also described in literature, including face, posterior neck, thigh, shoulder, and upper extremities [4, 5]. Accessory breast tissue in these atypical locations is partly explained by the second hypothesis, that is, from the modified apocrine sweat glands. Accessory breast tissue can occur with or without a nipple–areola complex. Clinically, it may be visible and palpable or very small and not palpable. In our case, breast tissue was not palpable in axilla, but the presence of fibroadenoma in the axilla indirectly indicates the existence of accessory breast. Moreover, its location in the axilla is in line with the first hypothesis.

Kajava developed the accessory breast classification system in 1915, which is still in use today [8, 11, 12] (Table 1). Since we only have a glandular tissue (detected as fibroadenoma) without any nipple–areola complex, our case belongs to class IV. Moreover, it is a rare occurrence of fibroadenoma in a nonpalpable axillary accessory breast tissue. The fibroadenoma originates from the nonpalpable accessory breast tissue located at the axilla in the milk line. Similar reports of fibroadenoma in a nonpalpable axillary accessory breast tissue were also put forward by Goyal *et al.* and Singh *et al.* [6, 13]

**Table 1:** Kajava classification

Type (class)	Description
Class I	Consists of a complete breast including glandular tissue, nipple, and areola
Class II	Consists of only glandular tissue and nipple, without areola
Class III	Consists of only glandular tissue and areola, without nipple
Class IV	Consists of only glandular tissue
Class V	Consists of only nipple and areola, without glandular tissue (pseudomamma)
Class VI	Consists of only the nipple (polythelia)
Class VII	Consists of only the areola (polythelia areolaris)
Class VIII	Consists of only hair (polythelia pilosa)

Cases of accessory breasts are usually sporadic, but familial presentations associated with urogenital and congenital cardiac abnormalities have been described, although the association has been challenged [14]. As stated specifically by some authors, patients with polythelia have been associated with urinary tract abnormalities such as supernumerary kidneys, failure of renal formation, renal adenocarcinoma, hydronephrosis, polycystic kidney disease, duplicate renal arteries, and ureteric stenosis. This association can be partly explained by the parallel development of mammary structure and genitourinary system [6, 8, 15]. Other congenital anomalies such as pyloric stenosis, epilepsy, and cardiac abnormalities are also seen [13]. Radiologic evaluation in our patient showed no urogenital or cardiac abnormalities. Accessory breast tissue is subject to hormonal response and may develop benign and malignant pathologic processes similar to those seen in normally located breast tissues, including fibrocystic disease, intraductal papilloma, lactating adenoma, fibroadenoma, and carcinoma [9, 10, 16]. Of these, carcinoma is reported as the common pathology [15].

Fibroadenoma is a benign biphasic tumor featuring a proliferation of both epithelial and stromal elements. It typically presents as a painless, firm, slow-growing, mobile, and well-defined breast mass. Fibroadenomas are among the most common benign tumors of the normally located breast in women under 30 years of age. In the adolescent population, the overall incidence of fibroadenoma is 2.2%. They account for 68% of all breast masses and 44–94% of biopsied breast lesions [17, 18].

Although fibroadenoma is a common benign lesion of normal breast tissue, its occurrence in accessory breast tissue is very rare and only few cases have been reported in the literature. Fewer than 40 cases of fibroadenoma in accessory breasts have been reported in the literature. In keeping with our case, the most common anatomic location was in the axilla, but it can occur anywhere in the milk line. However, cases have also been reported in other locations, including perineum, face, and back [4, 6, 19].

Differential diagnoses for a mass in the axillary region include lymphadenopathy (due to neoplastic, inflammatory, or infectious etiology), lipoma, neuroma, skin lesions (various cystic and tumorous lesions), axillary tail of Spence, and accessory breast tissue and lesions arising from it. In our case, the clinical impression was a lymphadenopathy, but FNAC suggested a fibroadenoma arising from accessory breast tissue, which was later confirmed by histopathology.

The diagnostic and therapeutic protocol for tumors in accessory breast tissue like in this case is similar to that of a normal breast mass. However, owing to its rarity and lack of suspicion, diagnosis may be delayed or even ignored, thus making timely treatment more difficult. When tumors or nodules are found along the mammary line, the presence of breast tissue should be considered during the investigation. As a quick and cost-effective procedure, FNAC is recommended to rule out the other differential diagnosis mentioned, to suggest the definitive diagnosis, and to guide the appropriate surgical intervention. [9, 15, 16, 20]

## Conclusion

For early diagnosis and management, accessory breast tissue disorders like fibroadenoma must be considered as a differential diagnosis while evaluating a patient with axillary swelling. This case underscores the fact that, similar to normal breast tissue, accessory breast tissue is also susceptible to the same physiologic and pathologic disease processes including neoplasms. Surgical intervention with excision is the mainstay of management, and FNAC can be used as a rapid and reliable procedure to facilitate early diagnosis. Moreover, genitourinary and cardiac evaluation for possible malformations is recommended.

## Data Availability

Data sharing does not apply to this article as no new data were created or analyzed in this study. **Ethics approval and consent to participate** Written informed consent for participation was obtained from the patient. We have also obtained ethical approval regarding the case from University of Gondar institutional review board. A copy of the consent form as well as ethical approval is available for review by the Editor of this journal.

## Abbreviations

ALT: Alanine transaminase
AST: Aspartate transaminase
BUN: Blood urea nitrogen
CBC: Complete blood count
ESR: Erythrocyte sedimentation rate
FNAC: Fine-needle aspiration cytology
MCV: Mean corpuscular volume
WBC: White blood cell
PO PRN: Per os pro re nata
